# Aging of TiO_2_ Nanoparticles Transiently Increases Their Toxicity to the Pelagic Microcrustacean *Daphnia magna*

**DOI:** 10.1371/journal.pone.0126021

**Published:** 2015-05-01

**Authors:** Frank Seitz, Simon Lüderwald, Ricki R. Rosenfeldt, Ralf Schulz, Mirco Bundschuh

**Affiliations:** 1 Institute for Environmental Sciences, University of Koblenz-Landau, Landau Germany; 2 Department of Aquatic Sciences and Assessment, Swedish University of Agricultural Sciences, Uppsala, Sweden; University of California, Merced, UNITED STATES

## Abstract

During their aquatic life cycle, nanoparticles are subject to environmentally driven surface modifications (e.g. agglomeration or coating) associated with aging. Although the ecotoxicological potential of nanoparticles might be affected by these processes, only limited information about the potential impact of aging is available. In this context, the present study investigated acute (96 h) and chronic (21 d) implications of systematically aged titanium dioxide nanoparticles (nTiO_2_; ~90 nm) on the standard test species *Daphnia magna* by following the respective test guidelines. The nTiO_2_ were aged for 0, 1, 3 and 6 d in media with varying ionic strengths (Milli-Q water: approx. 0.00 mmol/L and ASTM: 9.25 mmol/L) in the presence or absence of natural organic matter (NOM). Irrespective of the other parameters, aging in Milli-Q did not change the acute toxicity relative to an unaged control. In contrast, 6 d aged nTiO_2_ in ASTM without NOM caused a fourfold decreased acute toxicity. Relative to the 0 d aged particles, nTiO_2_ aged for 1 and 3 d in ASTM with NOM, which is the most environmentally-relevant setup used here, significantly increased acute toxicity (by approximately 30%), while a toxicity reduction (60%) was observed for 6 d aged nTiO_2_. Comparable patterns were observed during the chronic experiments. A likely explanation for this phenomenon is that the aging of nTiO_2_ increases the particle size at the start of the experiment or the time of the water exchange from <100 nm to approximately 500 nm, which is the optimal size range to be taken up by filter feeding *D*. *magna*. If subjected to further agglomeration, larger nTiO_2_ particles, however, cannot be retained by the daphnids’ filter apparatus ultimately reducing their ecotoxicological potential. This non-linear pattern of increasing and decreasing nTiO_2_ related toxicity over the aging duration, highlights the knowledge gap regarding the underlying mechanisms and processes. This understanding seems, however, fundamental to predict the risks of nanoparticles in the field.

## Introduction

The enormous production of engineered nanoparticles is suggested to contribute trillions of dollars to the global economy [1]. This high production comes along with their increasing use [2], which inescapably leads to their release into aquatic ecosystems via, for instance, wastewater treatment plant effluents [3]. On their way into as well as within aquatic ecosystems, nanoparticles are subject to environmentally driven modifications of their surface characteristics (e.g. size, surface area or charge) over time (= aging), which potentially alter their fate and toxicity. In this context, aging can include agglomeration and also coating of the particles’ surface with omnipresent natural organic matter (NOM) [4]. These processes are, for instance, triggered by the ionic strength and the quantity of NOM in the medium. In detail, high cation levels (high ionic strength) increase agglomeration speed [5], whereas NOMs stabilize or even disagglomerate particles [6] by inducing electrostatic repulsion [7].

Although these modifications are inevitable during the aquatic lifecycle of such engineered nanoparticles, the resulting modification of their ecotoxicological potential is largely unknown. Only a few studies have documented the acute [8,9] or chronic [10] effects of aged nanoparticles, and the single chronic experiment considered only one particular aging condition [10], hampering extrapolation of the findings. To overcome this limitation, the present study systematically varied the properties of the medium during the aging of the nanoparticles, prior to testing their acute and chronic ecotoxicological potential. In the context of the present study, the standard test organism *Daphnia magna* was used as model species to assess for both acute and chronic effects, while titanium dioxide nanoparticles (nTiO_2_, ~90 nm, ~99% anatase) served as model nanoparticles. This selection was motivated by (i) their frequent application in various products [2], (ii) the relatively good characterization of the potential effects on aquatic life (in particular *D*. *magna*) in an unaged form (e.g. [11,12]) as well as (iii) their potential to cause adverse effects in aquatic organisms at environmentally relevant concentrations [13].

It was hypothesized that higher ionic strength [14], which is considered as representative for natural freshwater environments, and the presence of a environmentally relevant NOM level [6,15] during aging as well as the longer duration of aging may decrease the nanoparticle-induced ecotoxicity to *D*. *magna*. Therefore, nTiO_2_ were aged for 0, 1, 3 and 6 d in media with varying ionic strength (Milli-Q water: approx. 0.00 mmol/L and ASTM: 9.25 mmol/L) with and without NOM and subsequently assessed for their acute toxicity (immobility). Based on these data, four scenarios were selected for the chronic studies, which showed particularly strong alterations in ecotoxicity. Accordingly, nTiO_2_ were aged for 0 or 3 d in ASTM with or without NOM prior to the evaluation of their respective chronic effects (reproduction, mortality) on daphnids. Both experimental setups were supplemented by particle size characterization before (acute toxicity tests) but also during the exposure (chronic) period.

## Material and Methods

### nTiO_2_ preparation and characterization

The titanium dioxide product A-100 (99% anatase) was provided as powder by Crenox GmbH (Germany), exhibiting an advertised primary particle size of 6 nm and a surface area of approximately 230 m^2^/g. Using this powder, a dispersant and additive free, size homogenized, stable suspension of ~90 nm was obtained by stirred media milling (PML 2, Bühler AG, Switzerland), and the resulting suspension was subsequently centrifuged (7500 rpm, ~20°C; Universal 320, Hettich, UK) in order to remove residual coarse material. Prior to its application the stock suspension (2 g nTiO_2_/L) was analysed for its particle size distribution (intensity weighted) using dynamic light scattering (n = 3 á 60 measurements; temperature: 20°C; pinhole: 100 μm; DelsaNano C, Beckman Coulter, Germany), which revealed a mean particle size of 87±1 nm (polydispersitiy index: 0.10–0.26). Moreover, before the start of each acute toxicity test and thus directly after (max. 3 min) the nTiO_2_ aging process (0, 1, 3, 6 d; Table 1) the mean initial particle size (particle size at the start of the experiment) was also determined in the respective aging medium. For all chronic studies the particle size was additionally monitored during the bioassay, on three consecutive days (representative of the time interval between the two water exchanges; Table 2). In order to exclude any measurement bias (e.g. a shifted particle size distribution induced by algal food, animal excrements), 3-mL samples were taken from one additional replicate without daphnids of a 2.00 mg nTiO_2_/L concentration at the center of the water column. Further, samples of test medium with NOM but without nTiO_2_ were analyzed to determine any background signals, which were not quantifiable. Moreover, during additional experiments the concentrations of aged nTiO_2_ were measured in the 4.00 mg/L treatment after 0 h and 96 h, representative of the start and the end of each acute toxicity test. For this purpose, inductively coupled plasma mass spectrometry (Table 3) was used according to methods described in detail by Rosenfeldt et al. [16]. As our chemical analysis revealed no substantial differences relative to the nominal concentrations, the present study is based on the nominal TiO_2_ concentrations exclusively.

**Table 1:** nTiO_2_ size after aging.

Aging medium	Acute toxicity tests							
	0 d		1 d		3 d		6 d	
	Initial particle size	PI^a^	Initial particle size	PI^a^	Initial particle size	PI^a^	Initial particle size	PI^a^
Milli-Q without NOM	82 (± 2)	0.11–0.23	81 (± 1)	0.13–0.19	82 (± 1)	0.16–0.17	81 (± 1)	0.16–0.17
Milli-Q with NOM	83 (± 1)	0.16–0.20	84 (± 1)	0.12–0.19	85 (± 1)	0.13–0.18	84 (± 1)	0.18–0.20
ASTM without NOM	1593 (± 53)	0.46–0.50	3712 (± 223)	0.89–1.00	2921 (± 103)	0.68–0.83	2530 (± 28)	0.67–0.74
ASTM with NOM	576 (± 11)	0.25–0.27	587 (± 27)	0.27–0.36	548 (± 11)	0.24–0.30	571 (± 10)	0.24–0.27

Mean initial particle size (± SD; n = 3) of nTiO_2_ aged for 0, 1, 3 and 6 d in different aging media with and without NOM (8 mg TOC/L), prior to its application in the respective acute toxicity test.

^a^Polydispersity index

**Table 2:** Particle size during chronic toxicity tests.

Aging conditions	Chronic toxicity tests					
Aging medium and duration (d)	0 d		1 d		2 d	
	Initial particle size	PI^a^	Initial particle size	PI^a^	Initial particle size	PI^a^
ASTM without NOM; 0	1334 (± 73)	0.30–0.41	1448 (± 90)	0.43–0.58	1453 (± 89)	0.48–0.60
ASTM without NOM; 3	5567 (± 709)	0.74–1.00	2992 (± 357)	0.42–1.00	2583 (± 380)	0.38–1.00
ASTM with NOM; 0	181 (± 68)	0.12–0.36	221 (± 46)	0.11–0.28	116 (± 1)	0.15–0.25
ASTM with NOM; 3	498 (± 36)	0.26–0.30	351 (± 21)	0.20–0.32	311 (± 14)	0.21–0.28

Mean particle size (± SD; n = 3) of aged nTiO_2_ (0 or 3 d) measured over 3 consecutive days (representative for the time between a water exchange) in the respective aging-/test medium, namely ASTM with and without NOM (8 mg TOC/L), during all chronic experiments.

^a^Polydispersity index

**Table 3:** Measured concentrations of nTiO_2_.

Aging conditions		Nominal concentration (mg/L)	Mean measured concentration (±SD; mg/L)	
Aging medium	Aging duration (d)	Test start 0 h	Test start 0 h	Test end 96 h
Milli-Q without NOM	0	4.00	3.82 ± 0.05	0.04 ± 0.00
	1	4.00	3.80 ± 0.07	0.04 ± 0.00
	3	4.00	4.02 ± 0.08	0.06 ± 0.00
	6	4.00	3.90 ± 0.24	0.04 ± 0.01
Milli-Q with NOM	0	4.00	3.71 ± 0.04	0.04 ± 0.01
	1	4.00	3.80 ± 0.04	0.05 ± 0.00
	3	4.00	3.80 ± 0.03	0.14 ± 0.01
	6	4.00	3.61 ± 0.05	0.05 ± 0.00
ASTM without NOM	0	4.00	3.57 ± 0.07	0.05 ± 0.00
	1	4.00	3.56 ± 0.07	0.05 ± 0.00
	3	4.00	3.57 ± 0.05	0.09 ± 0.00
	6	4.00	3.43 ± 0.06	0.05 ± 0.00
ASTM with NOM	0	4.00	3.59 ± 0.06	2.59 ± 0.04
	1	4.00	3.60 ± 0.04	3.28 ± 0.05
	3	4.00	3.54 ± 0.05	3.41 ± 0.06
	6	4.00	3.42 ± 0.02	3.21 ± 0.06

Nominal and mean measured (± SD; n = 3) nTiO_2_ concentrations after 0, 1, 3 and 6 d aging in the respective aging medium, namely ASTM with and without NOM (8 mg TOC/L).

### Test organism

*Daphnia magna* (Eurofins-GAB, Germany) were kept in permanent culture within a climate controlled chamber (Weiss Environmental Technology Inc., Germany) at 20±1°C with a 16:8 h (light:dark) photoperiod (visible light intensity, 3.14 W/m^2^; UVA, 0.109 W/m^2^; UVB, 0.01 W/m^2^). Thereby, groups of 25 organisms were cultured in 1.5 L of reconstituted hard freshwater (= ASTM) according to the ASTM International standard guide E729 [17]. The medium was additionally enriched with selenium, vitamins (thiamine hydrochloride, cyanocobalamine, biotine) and seaweed extract (Marinure, Glenside, Scotland; cf. [18]) and was renewed three times a week. Animals were fed on a daily basis with the green algae *Desmodesmus* sp. (200 μg C per organism).

### nTiO_2_ aging process

Prior to the start of any bioassay, nTiO_2_ were aged for different time intervals, i.e., 0, 1, 3 or 6 d (acute toxicity tests) and 0 or 3 d (chronic toxicity tests). Either Milli-Q water (solely used for the nTiO_2_ aging prior to the acute toxicity tests) or ASTM (as part of both: acute and chronic toxicity tests) was used as aging medium. The first represents a medium without ions (Milli-Q; nominally: 0.00 mmol/L) and the second a comparably high ionic strength (ASTM; nominally: 9.25 mmol/L; S1 Table). Additionally, for both media, the absence and presence of NOM was obtained using seaweed extract addition (cp. section: test organism) at concentrations of 0.0 or 8.0 mg TOC/L. The selection of this organic matter was based on (i) its recommendation as a medium additive during chronic metal toxicity tests with *Daphnia* [19,20] and (ii) its relatively balanced composition in terms of chromophoric dissolved organic carbon, which is also representative for NOM released from waste water treatment plants (S2 Table). The aging of nTiO_2_ in Milli-Q (with and without NOM) took place in 15 mL centrifuge vials with a nominal concentration of 1.00 g nTiO_2_/L. In contrast, the nTiO_2_ (nominal concentrations: 0.00–128.00 mg nTiO_2_/L) aging in ASTM with and without NOM was accomplished in a 500 mL glass vessel. Independent of the medium, each aging process was performed in total darkness (excluding photoactivation of nTiO_2_ to avoid the oxidation of NOM during aging) on a horizontal shaker (50 rpm; VKS-B-50, Edmund Bühler GmbH, Germany). Prior to toxicity testing all aged and unaged suspensions were vortexed for 30 seconds ensuring a homogenous distribution of nTiO_2_ (Table 3).

### Acute toxicity tests

During all acute toxicity tests, groups (n = 4) of five juvenile (<24 h) daphnids each were exposed for 96 h to different concentrations of 0, 1, 3 or 6 d aged nTiO_2_. Each acute toxicity test was conducted according to the OECD guideline 202 [21], during which daphnids were checked for immobilization every 24 h. In a first experiment (nTiO_2_ aged in Milli-Q with and without NOM), measured amounts of the aged and unaged nTiO_2_ stock suspension were added to ASTM (without NOM), resulting in a series of nominal nTiO_2_ concentrations with 0.0 (= control), 0.5, 1.0, 2.0, 4.0, 8.0 and 16.0 mg/L. Subsequently, daphnids were carefully transferred to 50 mL of each treatment. In contrast, for experiments with nTiO_2_ aged in ASTM with and without NOM, juvenile daphnids were directly placed in the aged and unaged nTiO_2_ ASTM suspensions, which were evenly distributed in 50 mL volumes (using concentrations from 0.00 (= control) to 128.00 mg nTiO_2_/L). All acute toxicity tests were conducted at 20±1°C with a 16:8 h (light:dark) photoperiod (visible light intensity, 3.14 W/m^2^; UVA, 0.109 W/m^2^; UVB, 0.01 W/m^2^).

### Chronic toxicity tests

Each chronic toxicity test was conducted according to the OECD guideline 211 [19]. Briefly, during all chronic toxicity tests, daphnids were exposed for 21 d to different nominal concentrations (i.e., 0.00 (= control), 0.02, 0.06, 0.20, 0.60, 2.00 or 6.00 mg/L) of 0 or 3 d aged nTiO_2_ using ASTM with and without NOM as aging medium. The aging duration was selected based on the outcome of the acute experiments, where nTiO_2_ aging for 3 d (ASTM with NOM) displayed an increased toxicity compared to unaged (0 d) nTiO_2_ in the presence of NOM. In detail, ten daphnids (<24 h) were individually placed in 50 mL of aged and unaged nTiO_2_ and fed daily with *Desmodesmus* sp. (from 50 to 100 μg C/organism with increasing age). Dead animals as well as released offspring were counted and removed daily. The test medium was renewed three times a week, while adult daphnids were carefully transferred using plastic pipettes. Dissolved oxygen (median: 7.8 mg/L) and pH (median: 8.2) fulfilled the requirements of the guideline [19] (S3 Table). Each chronic toxicity test was—similar to the acute toxicity tests—conducted at 20±1°C with a 16:8 h (light:dark) photoperiod (visible light intensity, 3.14 W/m^2^; UVA, 0.109 W/m^2^; UVB, 0.01 W/m^2^).

### Statistical analysis

Acute toxicity of differently aged nTiO_2_ suspensions was analyzed for the respective 96-h EC_50_ values (concentration at which half of the tested organisms are affected). Therefore, immobilization data of each acute toxicity test was corrected for control mortality (never exceeding 20%) using Abbott's formula. Subsequently, adequate dose-response models were fitted to these data (S1, S2, S3 and S4 Figs). The model selection was based on Akaike's information criterion and expert judgment (S4 Table). Finally, EC_50_ values were assessed for statistically significant differences among aging conditions using confidence interval testing [22].

For each chronic reproduction test, the cumulative mean offspring (after 21 d) was calculated separately (considering each treatment and aging process). Afterwards, differences in effect sizes (for the control and the highest nTiO_2_ concentrations, respectively; i.e., 2.00 and 6.00 mg nTiO_2_/L) among the different aging processes were statistically compared also using confidence interval testing [23]. Higher numbers indicate a higher effect size in terms of a decreased cumulative reproduction relative to the control. Additionally, a time to event analysis was accomplished by separately applying the Kaplan-Meier estimator for the data of each aging condition at the highest nTiO_2_ concentrations (2.00 or 6.00 mg/L). For all statistical analyses and figures the statistical software environment R for Windows [24] and corresponding packages [25,26,27] were used.

## Results

### Toxicity of aged and unaged nTiO_2_: absence of NOM

**Fig 1:**
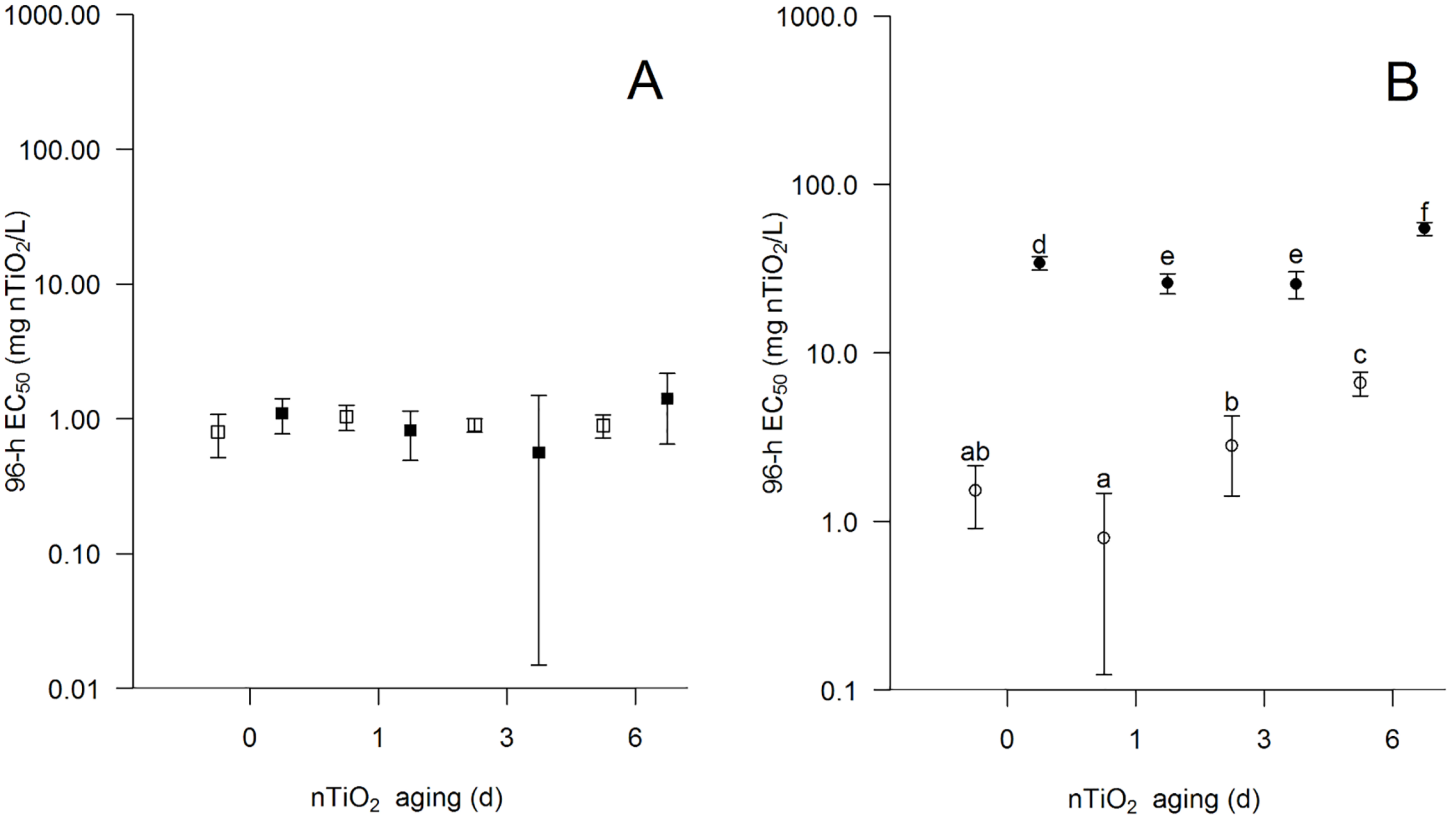
Acute toxicity of aged nTiO2. (A) 96-h EC50 values (half maximal effective concentration; ± 95% CI) of nTiO2 aged for 0, 1, 3 or 6 d in Milli-Q with (■) or without (□) NOM. (B) 96-h EC50 values (± 95% CI) of nTiO2 previously aged for 0, 1, 3 or 6 d in ASTM with (●) and without (○) NOM. 96-h EC50 values followed by different lower case letters are significantly different.

**Fig 2:**
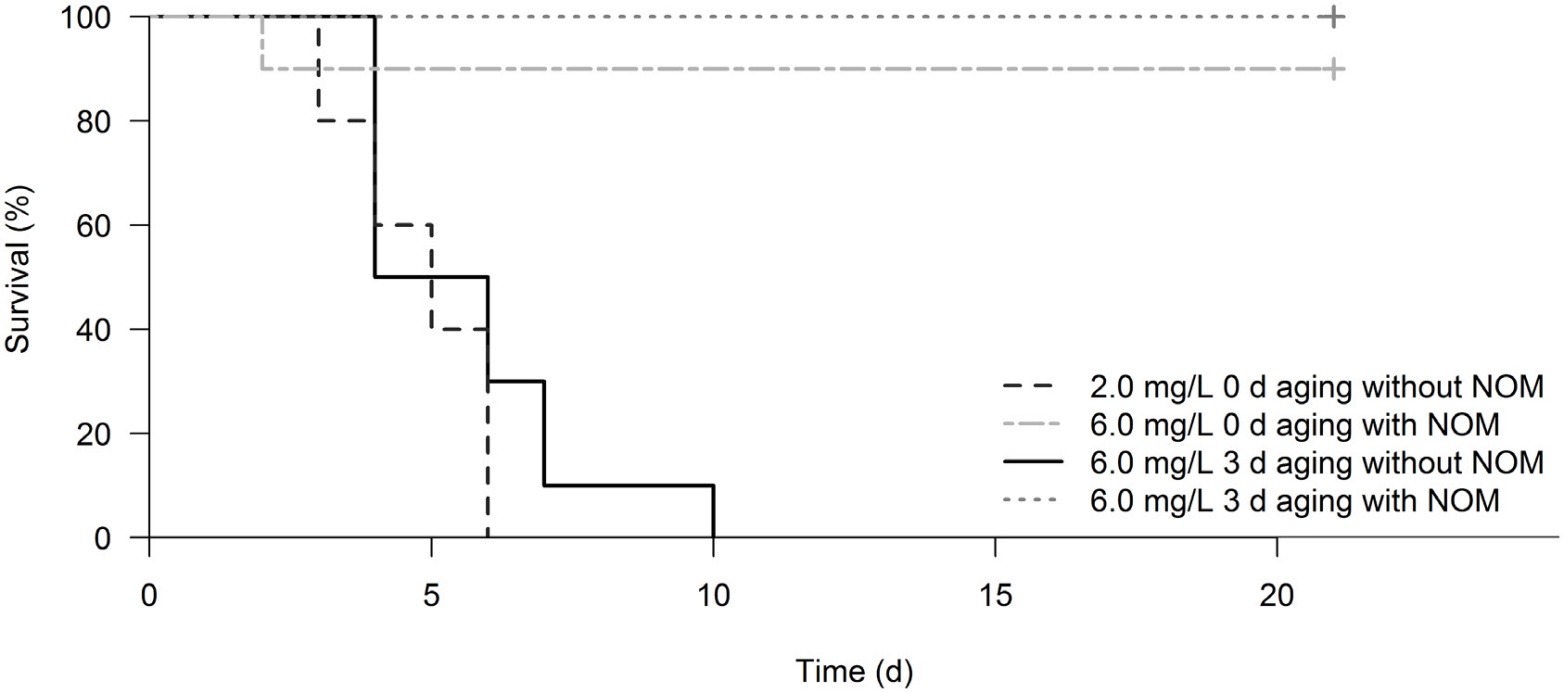
Survival time analysis during chronic exposure. Survival (%) of daphnids during the 21 d chronic toxicity tests with nTiO_**2**_. Lines represent the exposure to nTiO_**2**_ (i.e. 2.0 or 6.0 mg/L) aged under varying conditions (0 or 3 d) in ASTM with and without NOM.

**Fig 3:**
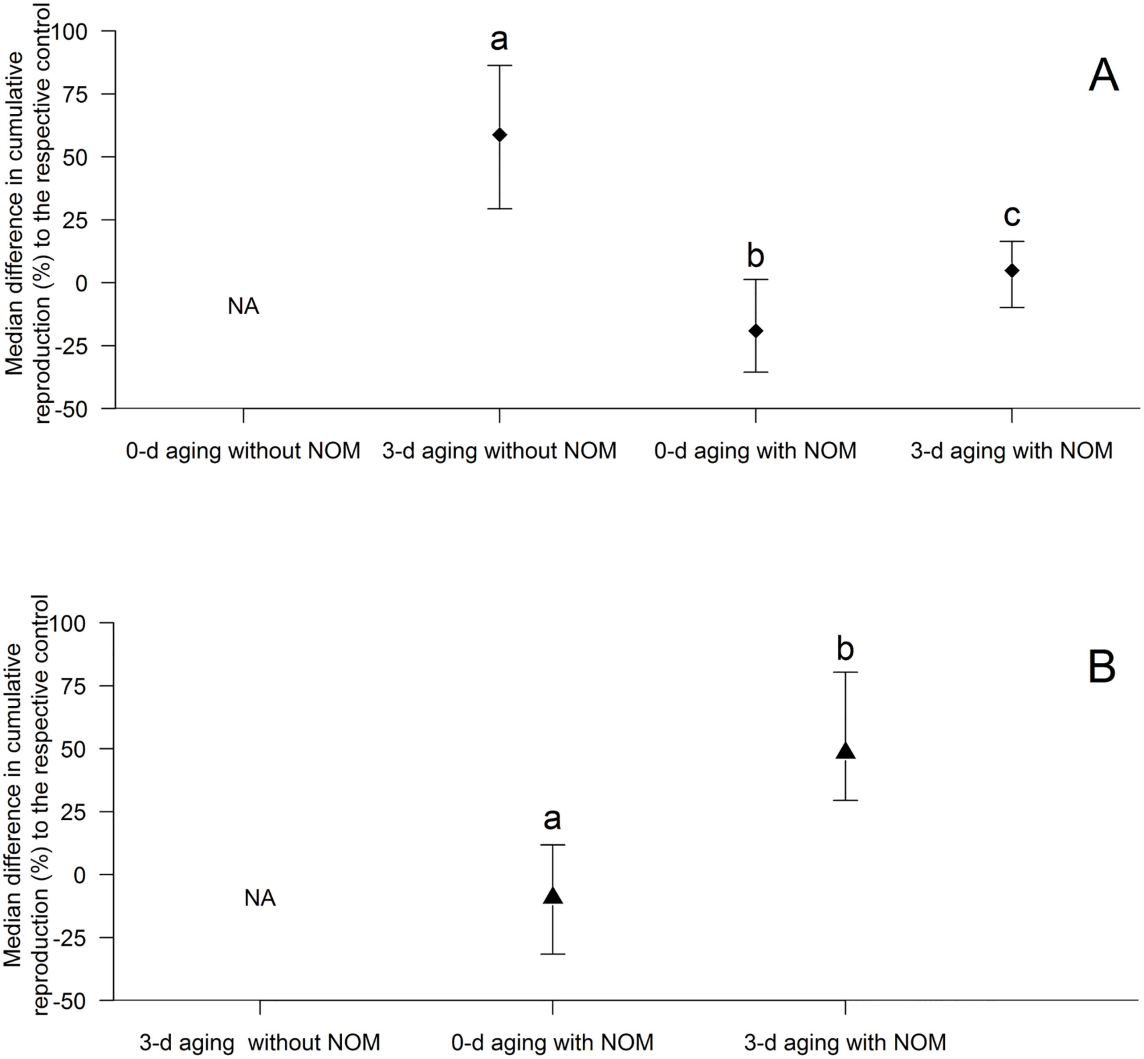
Fecundity of *Daphnia*. Median difference in the reproduction of *Daphnia* (± 95% CI) after 21 d of exposure to (A) 2.0 and (B) 6.0 mg nTiO_**2**_/L expressed relative to the respective control containing 0.0 mg/L nTiO_**2**_. Higher numbers indicate a decreased cumulative reproduction compared to the control. NA = not assessed due to 100% mortality in the nTiO_**2**_ treatment. Values followed by different lower case letters denote a statistically significant difference.

We detected similar 96-h EC_50_ outcomes (0.9 ‒ 1.4 mg nTiO_2_/L; Fig 1A) when using Milli-Q without NOM as an aging medium, independent of the applied aging duration. Aging of nTiO_2_ in ASTM without NOM revealed a lower ecotoxicity compared to Milli-Q without NOM (up to 7.5-fold; Fig 1A and 1B). Further, the nTiO_2_ toxicity decisively dropped for a 3 and 6 d aging (~1.7 and ~4-fold; Fig 1B; S5 Fig), when compared to the 0 d aging in ASTM without NOM. Similarly, 3 d aging of nTiO_2_ in ASTM without NOM reduced the chronic toxicity. In detail, 2.00 mg/L of 0 d aged nTiO_2_ caused 100% mortality in daphnids after six days of exposure (Fig 2) and thus there was no reproductive outcome. In contrast, at the same concentration of nTiO_2_ but aged for 3 d the mortality dropped to only 50% at the termination of the experiment (S6 Fig) accompanied by an approximately 60% reduced fecundity compared to the respective control (Fig 3A).

### Toxicity of aged and unaged nTiO_2_: presence of NOM

When aging nTiO_2_ in Milli-Q with NOM, no statistical significant change in the acute toxicity became evident relative to the unaged control and the aging in Milli-Q without NOM (Fig 1A). In contrast, a 0, 1, 3 or 6 d aging of nTiO_2_ in ASTM with NOM, revealed a significantly lower acute toxicity (8 to 33-fold; Fig 1B, see also S5 Fig) when compared to the aging in ASTM without NOM. This reduction in toxicity was also observed during the chronic experiment (ASTM with and without NOM). For instance, a 0 and 3 d aging of 2.00 and 6.00 mg/L nTiO_2_ in ASTM without NOM displayed 100% mortality of daphnids after 21 days (S6A and S6B Figs), whereas the aging in presence of NOM led to a reduced mortality, which is—depending on the nTiO_2_ concentration—equal to or below 10% (Fig 2). Moreover, while the cumulative reproduction of *Daphnia* was significantly reduced (~60%; Bonferroni adjusted pairwise Wilcoxon rank sum test: p = 0.027) at 2.00 mg/L of 3 d aged nTiO_2_ in ASTM without NOM when compared to the respective control, no reproductive implications became evident for the same concentration using ASTM with NOM as aging medium (Bonferroni adjusted pairwise Wilcoxon rank sum test: p = 1; Fig 3A).

When aged in ASTM with NOM, the acute toxicity of nTiO_2_ displayed a nonlinear pattern: relatively short aging durations of 1 or 3 d increased the acute toxicity of nTiO_2_ by up to 27% relative to the 0 d scenario (Fig 1B), whereas an extension of the aging duration to 6 d significantly reduced the acute nTiO_2_ toxicity (~two-fold) relative to all other scenarios (Fig 1B). In accordance with the acute toxicity data, chronic toxicity also increased for 3 d aged nTiO_2_ in ASTM with NOM (Fig 3A and 3B). In detail, an approximately 50% decline in reproduction of *Daphnia* was observed at 6.00 mg nTiO_2_/L aged for 3 d compared to the respective control (Fig 3B; S7D Fig, Bonferroni adjusted pairwise Wilcoxon rank sum test; p = 0.003). In contrast, the same concentration of 0 d aged nTiO_2_ did not affect the animals' reproduction significantly (Fig 3B; S7C Fig; Dunnett test: p = 0.554). Moreover, a direct comparison of both scenarios (i.e. 0 d aging vs 3 d aging) revealed a 60% higher effect size for a 3 d aging (confidence interval testing: p<0.05; Fig 3B).

## Discussion

### Toxicity of aged and unaged nTiO_2_: absence of NOM

Results of our acute toxicity tests showed that nTiO_2_ aged in Milli-Q without NOM did not influence the nanoparticles toxicity even after elongated aging periods (1, 3 and 6 d) and thus revealed comparable 96-h EC_50_ values for *Daphnia*'s immobility (Fig 1A). These results can be attributed to largely unchanged nTiO_2_ characteristics after aging in Milli-Q without NOM. In particular, the nTiO_2_ initial size ‒ which has been suggested as an important factor driving the extent of nanoparticle toxicity [28,29] ‒ was similar to the original nTiO_2_ stock solution irrespective of the aging duration (see Table 1). These observations may be attributed to a lack of ions during aging (ionic strength: approx. 0.00 mmol/L), which accelerates nTiO_2_ agglomeration in liquid media [30].

The importance of ions in the medium is also supported by the results of the present study. In contrast to the stable toxicity of nTiO_2_ aged in Milli-Q (aging medium of low ionic strength), nanoparticles aged in ASTM (aging medium of high ionic strength) showed a significant shift in their toxicity with aging duration. In particular, acute as well as chronic toxicity of nTiO_2_ aged in ASTM without NOM decreased with increasing aging duration (Fig 1B; Fig 2, Fig 3A and 3B). This reduced toxicity may be explained by altered nTiO_2_ characteristics, particularly the particle size at the initiation of the exposure of daphnids (Table 1). In other words, the relatively high ecotoxicity of 0 and 1 d aged particles can be associated with a potentially higher share of small sized nTiO_2_ in the water phase (see [11,28]) when compared to longer aging periods. As a result of the elongated aging duration, the particle size at the test initiation increases (e.g. after 6 d aging; ~2500 nm; Table 1) facilitating a fast sedimentation of nTiO_2_ agglomerates (as visually observed) and hence reduction of the nTiO_2_ concentration in the water column (the location where daphnids mainly dwell; Table 3). Moreover, such bigger particles are less likely to coat the surface of daphnids outer shell, a mode of toxic action of these nanoparticles suggested by Dabrunz et al. [11]. This in turn might affect the test species molting success [11] as well as their movement [31] and ultimately the mortality of *Daphnia*.

### Toxicity of aged and unaged nTiO_2_: presence of NOM

The particle size of nTiO_2_ at the initiation of the bioassay did not change with aging duration in Milli-Q with NOM medium (Table 1), which probably also explains the absence of any difference in the EC_50_ values relative to nTiO_2_ aged in Milli-Q without NOM (Fig 1A). The rather stable particle sizes over 6 d of aging in Milli-Q with NOM can be attributed to the low ion concentration (ionic strength: approx. 0.00 mmol/L) together with a NOM-induced particle size stabilization. In contrast to an aging in Milli-Q with NOM, the aging in ASTM with NOM generally reduced the acute and chronic toxicity of nanoparticles relative to ASTM without NOM (Fig 1B, Fig 2, Fig 3A and 3B). This result may directly be related to NOM coating both the nanoparticles (indicated by an decreased zeta potential of nTiO_2_; see [32]) and the test organisms [33]; coating which was largely absent for nTiO_2_ aged in Milli-Q with NOM due to the lower NOM concentrations in the test medium. In ASTM with NOM, the electrosteric repulsion [34] kept nTiO_2_ in the water phase and prevented an attachment to the surface of *Daphnia* [33]. In addition, NOM coating may have limited irradiation of the nanoparticle surfaces and potentially scavenged harmful reactive oxygen species [35], which are usually formed by nTiO_2_ under the visible light conditions in our experimental facilities [36]. Moreover, the NOM may have served as a energy source for *Daphnia* [37], which may have lowered the overall toxicity of aged and unaged nTiO_2_ indirectly as a result of an increased fitness of the test organisms [38]. This assumption is also supported by an approximately 40% higher reproductive output of daphnids cultured under control conditions but in presence of NOM relative to its absence (S7A and S7C Figs).

**Fig 4:**
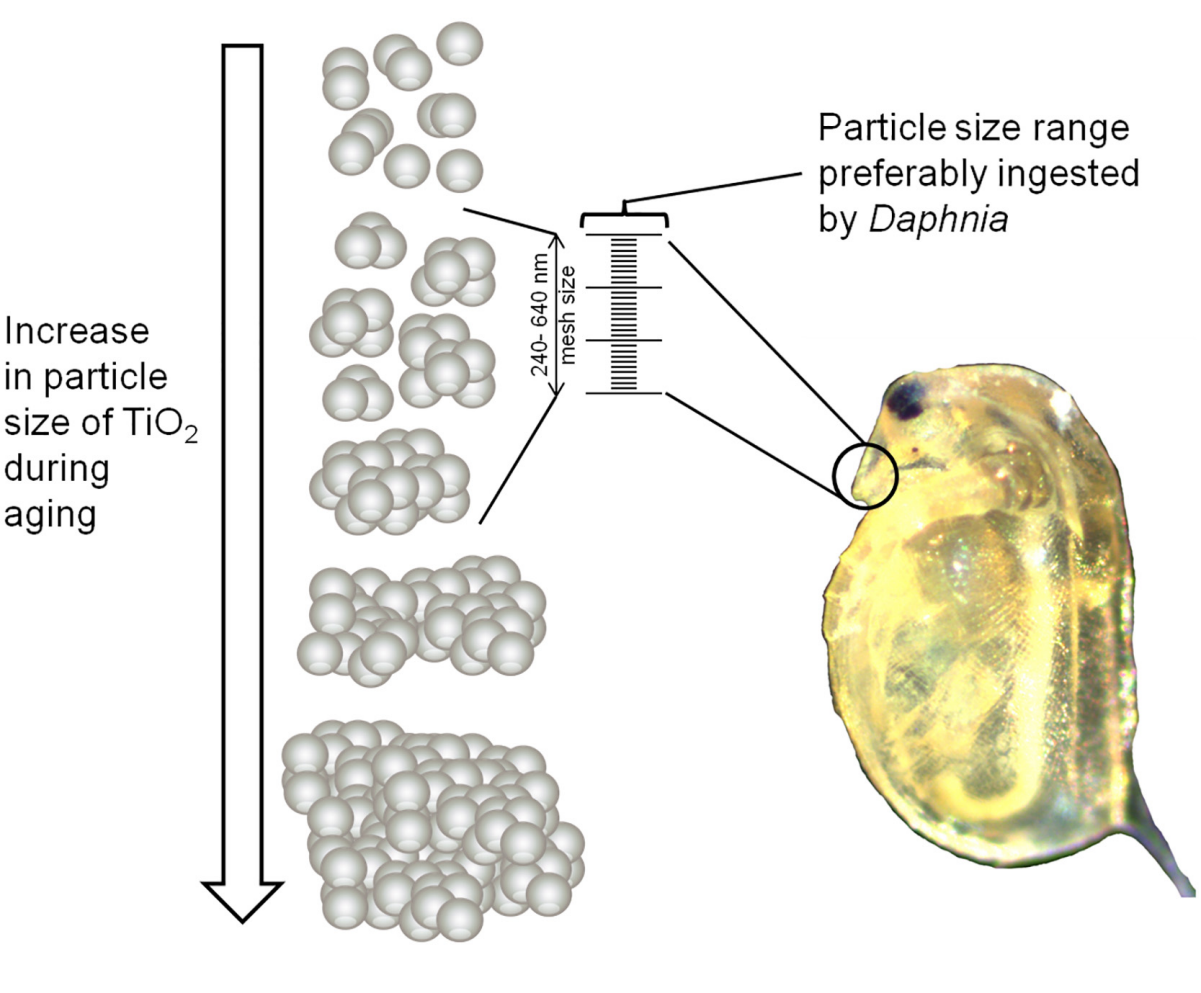
Particle uptake of *Daphnia*. Schematic draft of the preferably ingested particle size range of *Daphnia magna*.

Irrespective of the general tendency of NOM to reduce the ecotoxicity of nTiO_2_, especially after 6 d of aging in ASTM with NOM, an aging of these particles for 1 and 3 d in the same medium induced an increased acute as well as chronic toxicity relative to the respective unaged particles (Fig 1B, Fig 2, Fig 3A and 3B). This pattern may be explained by (i) a relatively high number of small and ecotoxicological potent particles ‒ while the predominance of this size fraction likely decreased with increasing aging duration as well as (ii) size stabilized nTiO_2_ agglomerates of ~500 nm size (due to NOM; Table 1). The smaller particles might have mainly contributed to the acute nTiO_2_ toxicity after 0 and 1 d of aging, while for 3 d aged nTiO_2_ bigger agglomerates may have induced adverse effects. The latter suggestion may be explained by the mesh size of *D*. *magna*'s filter apparatus ‒ displaying a range of 0.24–0.64 μm [39] ‒ which facilitates an uptake of ~500 nm agglomerates (in sensu [40,41]; Fig 4). However, the size range of the organisms filter apparatus also indicates that a limited amount of smaller particles of unaged nTiO_2_ (with a mean particle size of approximately 180 nm; Table 2) were actively ingested by the test species (due to their filter passage). Hence, a higher mass of nTiO_2_ might have been taken up by daphnids if exposed to nTiO_2_ aged for 3 d (mean particle size: ~500 nm; ASTM with NOM; Table 2) relative to the same product aged for 0 d (mean particle size: ~180 nm; Table 2). During the acute experiments this hypothesized increased accumulation of nTiO_2_ in the gut [42,43] may have reduced their mobility [31] ultimately increasing the mortality (immobility) of daphnids. Similarly, during the chronic experiments such nTiO_2_ agglomerates may have decisively lowered the amount of ingested algae (cf. [40,43]) limiting at the same time the energy availability for daphnids. Such implications in the energy processing might have led to a decreased fecundity [44] during the chronic investigations at nTiO_2_ concentrations as high as 6.00 mg/L (Fig 3B). This is further supported by findings of previous investigations [13,18] where, nTiO_2_ agglomerates of ~330 nm revealed statistically significant implications in *Daphnia’s* reproduction output [18], while smaller agglomerates (~140 nm) did not affect the animals fecundity [13]. However, our findings are widely contrary to the common scientific assumption (especially when considering results obtained with nTiO_2_ aged for up to 3d), which expects stable or decreased toxicity of nanoparticles with aging duration and thus elevating agglomeration and sedimentation [8,14,45,46].

Results of the present study thus showed that the aging duration as well as the properties of the medium (in terms of ionic strength and NOM content) alter the nTiO_2_-related toxicity. As the effect-size and-direction strongly varied, the hypothesis, that aging reduces nTiO_2_ toxicity, is not completely supported. Although it is obvious that the presence of NOM reduced the toxicity of nanoparticles substantially, the ultimate risk associated with their release may by underestimated when ignoring the aging history. Since similar patterns may also be relevant for other types of nanoparticles, it seems sensible to uncover the underlying mechanisms and assess for their transferability among different classes of nanoparticles. In the light of recent literature and the present study, it seems moreover crucial to consider the implications of environmental parameters such as differing ionic strengths (representing for instance fresh- and seawater) and NOM levels (being present in nature)—on the ecotoxicological potential of nTiO_2_ in particular and nanoparticles in general. Moreover this may also account for ultraviolet irradiation, which likely potentiates negative effects [47]. Overall, the present study provides an example on how nanoparticle-typical environmental processes, such as agglomeration during aging, lead to a varying toxicity profile over time, which may be explained when coupled with ecological information, such as particle size selectivity of *Daphnias’* filtering apparatus.

## Supporting Information

S1 Fig Dose-Response curves underlying the 96-h EC_50_ calculations for (A) 0, (B) 1, (C) 3 and (D) 6 d aged nTiO_2_ in Milli-Q without NOM. The mean mortality for each treatment is indicated by an open circle, while the filled circle represents the EC_50_ value together with its 95% confidence interval.
(PDF)

S2 Fig Dose-Response curves underlying the 96-h EC_50_ calculations for (A) 0, (B) 1, (C) 3 and (D) 6 d aged nTiO_2_ in Milli-Q with NOM. The mean mortality for each treatment is indicated by an open circle, while the filled circle represents the EC_50_ value together with its 95% confidence interval.
(PDF)

S3 Fig Dose-Response curves underlying the 96-h EC_50_ calculations for (A) 0, (B) 1, (C) 3 and (D) 6 d aged nTiO_2_ in ASTM without NOM. The mean mortality for each treatment is indicated by an open circle, while the filled circle represents the EC_50_ value together with its 95% confidence interval.
(PDF)

S4 Fig Dose-Response curves underlying the 96-h EC_50_ calculations for (A) 0, (B) 1, (C) 3 and (D) 6 d aged nTiO_2_ in ASTM with NOM. The mean mortality for each treatment is indicated by an open circle, while the filled circle represents the EC_50_ value together with its 95% confidence interval.
(PDF)

S5 Fig 96-h EC_50_ values (half maximal effective concentration; ± 95% confidence interval) of nTiO_2_ previously aged for 0, 1, 3 or 6 d in ASTM with (●) and without (○) NOM. Asterisk (*) denotes statistical significant difference to the respective 96-h EC_50_ value. (PDF)

S6 Fig Survival (%) of daphnids during 21 d of nTiO_2_ exposure. Different lines represent response of *Daphnia* in the respective nTiO_2_ treatment—the concentrations are indicated in the legend of each figure. (A) Animals exposed to nTiO_2_ aged for 0 d in ASTM without NOM. (B) Animals exposed to nTiO_2_ aged for 3 d in ASTM without NOM. (C) Animals exposed to nTiO_2_ aged for 0 d in ASTM with NOM. (D) Animals exposed to nTiO_2_ aged for 3 d in ASTM with NOM.
(PDF)

S7 Fig Cumulative median (± SD) reproduction per test-organism after 21 d of exposure to differently aged nTiO_2_. (A) Animals exposed to nTiO_2_ aged for 0 d in ASTM without NOM (○). (B) Animals exposed to nTiO_2_ aged for 3 d in ASTM without NOM (□). (C) Animals exposed to nTiO_2_ aged for 0 d in ASTM with NOM (●). (D) Animals exposed to nTiO_2_ aged for 3 d in ASTM with NOM (■). Asterisks denote statistical significant difference relative to the respective control; p < 0.05 (*), p < 0.01 (**). NA indicates a not assessable reproductive output due to 100% mortality of adult daphnids in the respective treatment.
(PDF)

S1 Table Composition and ionic strength of the ASTM test medium. (PDF)

S2 Table Dissolved organic carbon analysis (μg/L-C; if not stated otherwise) for seaweed extract (SW) applying SEC-OCD-OND (size-exclusion chromatography—organic carbon detection—organic nitrogen detection. (PDF)

S3 Table Mean (± SE; n = 3) water quality parameters measured over the entire test duration of each *Daphnia* reproduction experiment. (PDF)

S4 Table Model specification and respective Akaike's information criterion on which each 96-h EC_50_ value is based. (PDF)
