# Supplementary material for: Aging of TiO2 Nanoparticles Transiently Increases Their Toxicity to the Pelagic Microcrustacean Daphnia magna
Source: PLoS One. 2015 May 1;10(5):e0126021. doi: 10.1371/journal.pone.0126021 (PMC4416768; doi:10.1371/journal.pone.0126021)

S4 Figure. Dose-Response curves underlying the 96-h EC_{50} calculations for (A) 0, (B) 1, (C) 3 and (D) 6 d aged $nTiO_2$ in ASTM with NOM. The mean mortality for each treatment is indicated by an open circle, while the filled circle represents the EC_{50} value together with its 95% confidence interval.

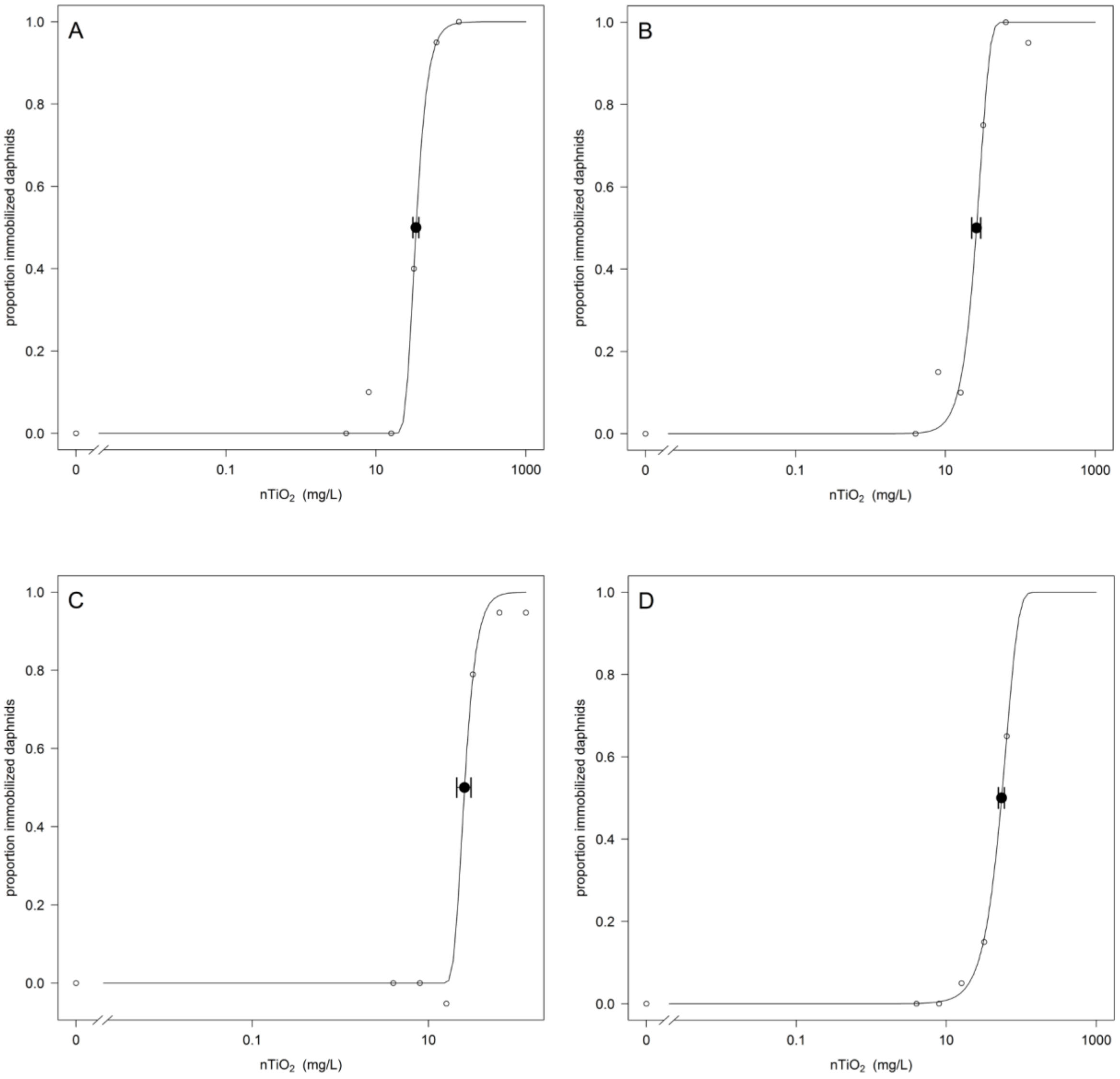

Supplement: S4 Fig — The mean mortality for each treatment is indicated by an open circle, while the filled circle represents the EC50 value together with its 95% confidence interval. (PDF) [file pone.0126021.s004.pdf]
