# Supplementary material for: Aging of TiO2 Nanoparticles Transiently Increases Their Toxicity to the Pelagic Microcrustacean Daphnia magna
Source: PLoS One. 2015 May 1;10(5):e0126021. doi: 10.1371/journal.pone.0126021 (PMC4416768; doi:10.1371/journal.pone.0126021)

S5 Figure. 96-h EC_{50} values (half maximal effective concentration; ± 95% confidence interval) of $nTiO_2$ previously aged for 0, 1, 3 or 6 d in ASTM with (●) and without (○) NOM. Asterisk (*****) denotes statistical significant difference to the respective 96-h EC_{50} value.

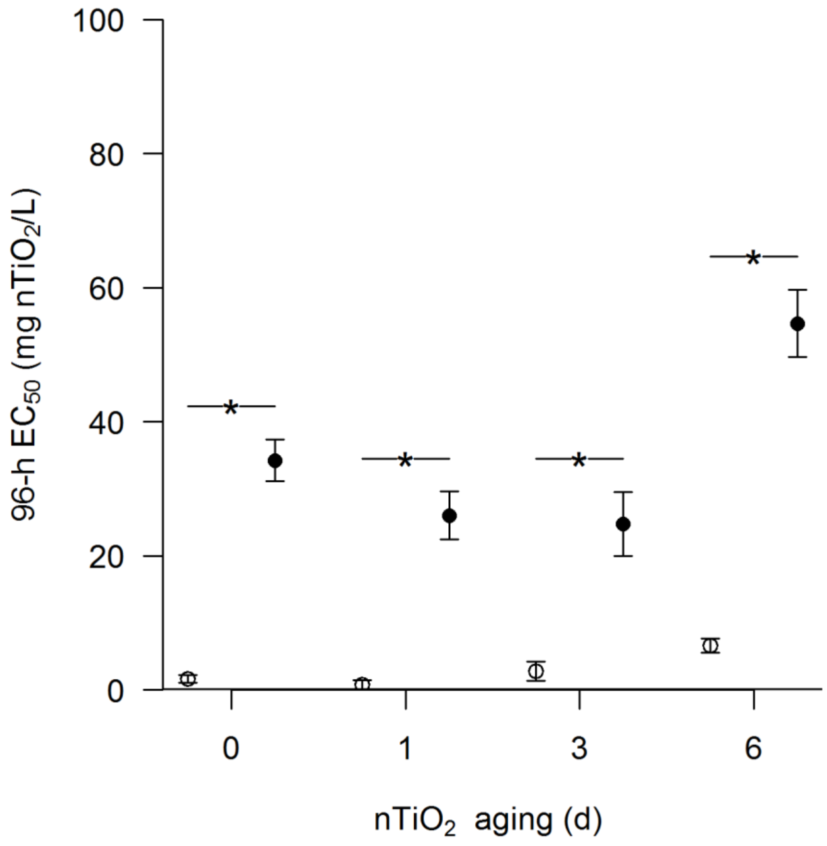

Supplement: S5 Fig — (PDF) [file pone.0126021.s005.pdf]
