# Supplementary material for: Aging of TiO2 Nanoparticles Transiently Increases Their Toxicity to the Pelagic Microcrustacean Daphnia magna
Source: PLoS One. 2015 May 1;10(5):e0126021. doi: 10.1371/journal.pone.0126021 (PMC4416768; doi:10.1371/journal.pone.0126021)

S6 Figure. Survival (%) of daphnids during 21 d of $nTiO_2$ exposure. Different lines represent response of *Daphnia* in the respective $nTiO_2$ treatment – the concentrations are indicated in the legend of each figure. (A) Animals exposed to $nTiO_2$ aged for 0 d in ASTM without NOM. (B) Animals exposed to $nTiO_2$ aged for 3 d in ASTM without NOM. (C) Animals exposed to $nTiO_2$ aged for 0 d in ASTM with NOM. (D) Animals exposed to $nTiO_2$ aged for 3 d in ASTM with NOM.

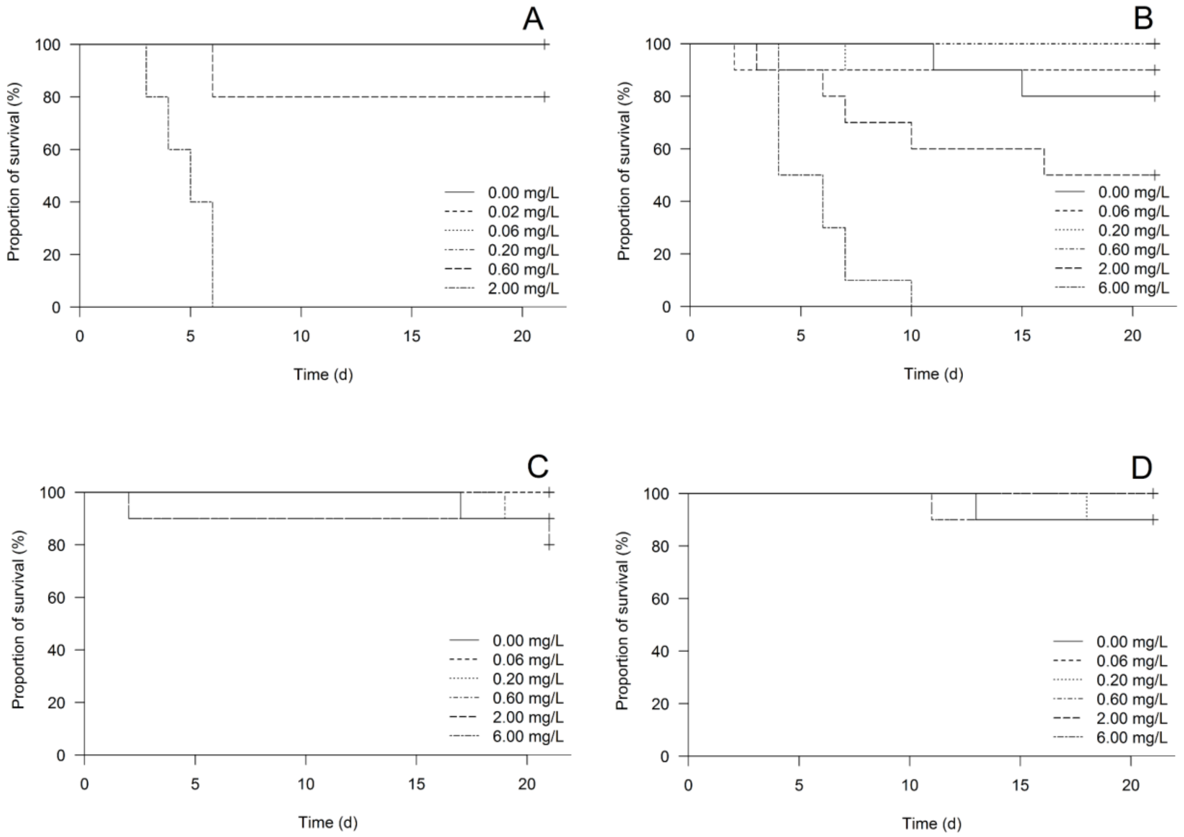

Supplement: S6 Fig — Different lines represent response of Daphnia in the respective nTiO2 treatment—the concentrations are indicated in the legend of each figure. (A) Animals exposed to nTiO2 aged for 0 d in ASTM without NOM. (B) Animals exposed to nTiO2 aged for 3 d in ASTM without NOM. (C) Animals exposed to nTiO2 aged for 0 d in ASTM with NOM. (D) Animals exposed to nTiO2 aged for 3 d in ASTM with NOM. (PDF) [file pone.0126021.s006.pdf]
