# Supplementary material for: Aging of TiO2 Nanoparticles Transiently Increases Their Toxicity to the Pelagic Microcrustacean Daphnia magna
Source: PLoS One. 2015 May 1;10(5):e0126021. doi: 10.1371/journal.pone.0126021 (PMC4416768; doi:10.1371/journal.pone.0126021)

S7 Figure. Cumulative median (± SD) reproduction per test-organism after 21 d of exposure to differently aged $nTiO_2$. (A) Animals exposed to $nTiO_2$ aged for 0 d in ASTM without NOM (○). (B) Animals exposed to $nTiO_2$ aged for 3 d in ASTM without NOM (□). (C) Animals exposed to $nTiO_2$ aged for 0 d in ASTM with NOM (●). (D) Animals exposed to $nTiO_2$ aged for 3 d in ASTM with NOM (■). Asterisks denote statistical significant difference relative to the respective control; $p < 0.05$ (*****), $p < 0.01$ (******). NA indicates a not assessable reproductive output due to 100% mortality of adult daphnids in the respective treatment.

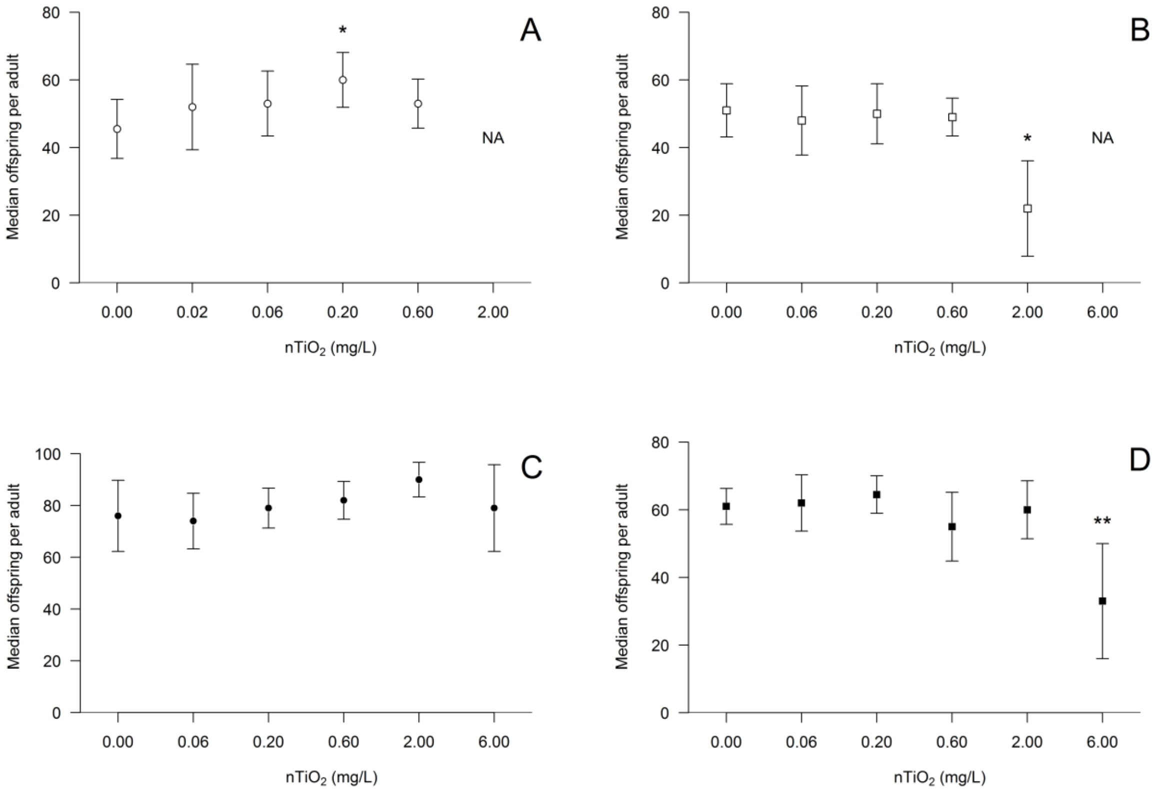

Supplement: S7 Fig — (A) Animals exposed to nTiO2 aged for 0 d in ASTM without NOM (○). (B) Animals exposed to nTiO2 aged for 3 d in ASTM without NOM (□). (C) Animals exposed to nTiO2 aged for 0 d in ASTM with NOM (●). (D) Animals exposed to nTiO2 aged for 3 d in ASTM with NOM (■). Asterisks denote statistical significant difference relative to the respective control; p < 0.05 (*), p < 0.01 (**). NA indicates a not assessable reproductive output due to 100% mortality of adult daphnids in the respective treatment. (PDF) [file pone.0126021.s007.pdf]
