# Supplementary material for: Aging of TiO2 Nanoparticles Transiently Increases Their Toxicity to the Pelagic Microcrustacean Daphnia magna
Source: PLoS One. 2015 May 1;10(5):e0126021. doi: 10.1371/journal.pone.0126021 (PMC4416768; doi:10.1371/journal.pone.0126021)
Supplement: S1 Table — (PDF) [file pone.0126021.s008.pdf]

S1 Table. Composition and ionic strength of the ASTM test medium.

Component	Concentration in mg/L
$NaHCO_3$	192
$CaSO_4 \cdot 2H_2O$	120
$MgSO_4$	120
KCl	8
Na_2SeO_3	0.00219
Thiamine hydrochloride (B_1)	0.075
Biotin (B_7)	0.00075
Cyanocobalamine (B_{12})	0.001
Ionic strength	9.25 mmol/L
