# Supplementary material for: Aging of TiO2 Nanoparticles Transiently Increases Their Toxicity to the Pelagic Microcrustacean Daphnia magna
Source: PLoS One. 2015 May 1;10(5):e0126021. doi: 10.1371/journal.pone.0126021 (PMC4416768; doi:10.1371/journal.pone.0126021)
Supplement: S2 Table — (PDF) [file pone.0126021.s009.pdf]

S2 Table. Dissolved organic carbon analysis (µg/L-C; if not stated otherwise) for seaweed extract (SW) applying SEC-OCD-OND (size-exclusion chromatography - organic carbon detection - organic nitrogen detection).

NOM	DOC										SUVA[a] (L/mg*m)
		HOC[b]	CDOC[c]								
	total			BIO-polymers	Humic Substance			Building Blocks	LMW[d] neutrals	LMW[c] acids	
		total	total	total	total	aromaticity (L/mg*m)	Mol-weight (g/mol)				
SW	1276	366	911	71	196	4.96	954	285	312	48	2.13

[a] specific UV absorbance; [b] hydrophobic organic carbon; [c] hydrophilic organic carbon; [d] low molecular weight
