# Supplementary material for: Aging of TiO2 Nanoparticles Transiently Increases Their Toxicity to the Pelagic Microcrustacean Daphnia magna
Source: PLoS One. 2015 May 1;10(5):e0126021. doi: 10.1371/journal.pone.0126021 (PMC4416768; doi:10.1371/journal.pone.0126021)
Supplement: S3 Table — (PDF) [file pone.0126021.s010.pdf]

S3 Table. Mean (± SE; n=3) water quality parameters measured over the entire test duration of each *Daphnia* reproduction experiment.

Aging conditions	1st Week			2nd Week			3rd Week		
	pH	Oxygen (mg/L)	Temperature (°C)	pH	Oxygen (mg/L)	Temperature (°C)	pH	Oxygen (mg/L)	Temperature (°C)
0 d aging ASTM without NOM	8.15 (± 0.03)	7.41 (± 0.60)	19.97 (± 0.09)	8.14 (± 0.05)	6.77 (± 1.03)	19.67 (± 0.27)	8.73 (± 0.30)	8.32 (± 0.11)	19.57 (± 0.24)
3 d aging ASTM without NOM	8.29 (± 0.01)	7.06 (± 0.21)	19.57 (± 0.23)	8.27 (± 0.08)	7.67 (± 0.66)	19.80 (± 0.26)	8.35 (± 0.08)	8.47 (± 0.12)	19.47 (± 0.20)
0 d aging ASTM with NOM	8.23 (± 0.04)	8.06 (± 0.35)	20.00 (± 0.31)	8.10 (± 0.03)	7.48 (± 0.26)	19.37 (± 0.32)	8.18 (± 0.02)	7.95 (± 0.09)	19.83 (± 0.09)
3 d aging ASTM with NOM	8.35 (± 0.00)	8.04 (± 0.12)	19.80 (± 0.12)	8.22 (± 0.05)	7.59 (± 0.11)	19.43 (± 0.48)	8.20 (± 0.01)	8.11 (± 0.09)	19.80 (± 0.12)
