# Supplementary material for: Aging of TiO2 Nanoparticles Transiently Increases Their Toxicity to the Pelagic Microcrustacean Daphnia magna
Source: PLoS One. 2015 May 1;10(5):e0126021. doi: 10.1371/journal.pone.0126021 (PMC4416768; doi:10.1371/journal.pone.0126021)
Supplement: S4 Table — (PDF) [file pone.0126021.s011.pdf]

S4 Table. Model specification and respective Akaike's information criterion on which each 96-h EC_{50} value is based.

Aging medium	Aging duration (d)	Model		Akaike's information criterion	Lack of fit
Milli-Q without NOM	0 d	two-parameter Weibull function	W1.2	-15.93	0.82
	1 d	two-parameter Weibull function	W2.2	-31.02	0.59
	3 d	two-parameter Weibull function	W2.2	-58.13	0.67
	6 d	two-parameter Weibull function	W2.2	-32.02	0.44
Milli-Q with NOM	0 d	log-normal dose-response model	LN.2	-31.62	0.40
	1 d	two-parameter Weibull function	W2.2	-21.28	0.96
	3 d	two-parameter Weibull function	W1.2	-5.68	0.84
	6 d	two-parameter Weibull function	W1.2	-6.49	0.46
ASTM without NOM	0 d	two-parameter Weibull function	W1.2	-34.85	0.98
	1 d	log-normal dose-response model	LN.2	-51.06	0.76
	3 d	two-parameter Weibull function	W1.2	-3.18	0.03
	6 d	two-parameter Weibull function	W2.2	-26.68	0.97
ASTM with NOM	0 d	two-parameter Weibull function	W1.2	-37.23	0.74
	1 d	two-parameter Weibull function	W2.2	-29.73	0.45
	3 d	two-parameter Weibull function	W1.2	-38.00	0.84
	6 d	two-parameter Weibull function	W2.2	-44.37	0.99
